# Classification of fielders in nippon professional baseball using a Gaussian mixture clustering model

**DOI:** 10.3389/fspor.2026.1612463

**Published:** 2026-02-19

**Authors:** Taishi Oda, Nobuyoshi Hirotsu

**Affiliations:** Graduate School of Health and Sports Science, Juntendo University, Bunkyō, Japan

**Keywords:** baseball, cluster analysis, Gaussian mixture model, principal components analysis, sabermetrics

## Abstract

**Summary:** This study proposes a novel analytical framework for categorizing Japanese professional baseball players based on comprehensive hitting performance data. Our primary goal is to identify player groupings that may inform decision-making related to substitution and trade strategies within teams. The dataset used in this analysis was provided by DELTA Corporation, a Japanese firm specializing in advanced baseball analytics. It includes 115 distinct hitting-related performance indices for 327 fielders who participated in official Nippon Professional Baseball (NPB) games during the 2020 season. To make the analysis more structured, we first organized these 115 indices into eight meaningful categories, following the classification methodology defined by DELTA. These categories represent various aspects of hitting performance, such as plate discipline, power, contact ability, and situational hitting, among others. To focus on players with a significant level of contribution, we filtered the original sample and selected 72 players who recorded a “Wins Above Replacement” (WAR) value of 1.0 or higher during the season.

## 1 Introduction

Baseball is a major sport in several countries, from amateur to professional levels. In Japan, twelve teams under the Nippon Professional Baseball (NPB) organization compete annually in official league games.

Statistical approaches to analyzing baseball performance, such as slugging percentage, on-base percentage, and OPS, are commonly referred to as sabermetrics (1). Within sabermetrics, multivariate analysis (2) has been widely adopted to examine the relationships among multiple performance indicators simultaneously.

Among multivariate techniques, cluster analysis and principal component analysis (PCA) have frequently been applied to classify athletes based on performance data. A representative example of cluster analysis is player classification using the k-means method (3), a non-hierarchical approach that partitions players into a predefined number of clusters based on cluster means. Tanaka et al. (4), for instance, classified collegiate baseball players according to swing characteristics measured by a swing analysis device and compared the clustering results with classifications based on batting performance.

However, k-means clustering requires the number of clusters to be specified in advance. In contrast, cluster analysis based on a Gaussian mixture model (GMM) can objectively determine the appropriate number of clusters using criteria such as the Bayesian Information Criterion (BIC) (5). Moreover, GMM-based clustering is well suited for multimodal data distributions, which often arise in sports performance data.

Sakaori et al. (6) applied GMM-based clustering to Japanese professional baseball pitchers from 2010 to 2014, using the number of pitches per game as the classification variable. They demonstrated that the pitch count distribution was multimodal and identified five distinct clusters based on BIC values. While this approach enabled objective classification, the cluster characteristics were determined solely by a single variable.

When cluster analysis is conducted using many variables, interpretation becomes increasingly difficult. When the number of variables exceeds approximately ten, identifying the defining characteristics of each cluster based on raw variable values becomes challenging. This limitation can be addressed by PCA (7), which reduces a large set of correlated variables to a smaller number of uncorrelated principal components while retaining most of the original information.

Kageyama et al. (8) applied PCA to seven swing-related variables of collegiate baseball players and successfully reduced them to two interpretable principal components representing spatial and temporal characteristics of the swing. This demonstrates that PCA can condense complex performance information into interpretable latent dimensions.

Building on these studies, this paper proposes a framework for player classification that integrates PCA and cluster analysis. Here, the term “framework” refers to the analytical flow in which high-dimensional performance variables are first condensed using PCA and subsequently classified using cluster analysis, rather than applying PCA or clustering independently. By performing cluster analysis on a reduced set of principal components, information loss from the original variables is minimized, and the interpretation of clustering results becomes more tractable.

A similar analytical flow was proposed by Soto-Valero et al. (9), who applied PCA to performance indices of 7,705 European soccer players and subsequently performed GMM-based clustering to identify groups of similar players. Nishiuchi (10) conducted PCA on more than 200 performance indices of J1 League soccer players and performed cluster analysis to generate eight clusters, suggesting that players within the same cluster could potentially substitute for one another.

In the present study, we extend this analytical framework to professional baseball players in Japan. While recent studies, such as Umemura (2025), have evaluated player trades in the NPB based on outcome-oriented metrics such as changes in WAR, our approach focuses instead on classifying players according to their latent performance characteristics. This perspective offers a complementary, structure-based view of player similarity.

Specifically, we apply PCA to the performance statistics of Japanese professional baseball fielders to reduce dimensionality and then classify players using a Gaussian mixture model. We examine whether players within the same cluster can be regarded as potential substitutes, and whether players from different clusters may represent plausible trade counterparts. By comparing our classification-based approach with outcome-based evaluations, we discuss the potential implications of this framework for roster construction, substitution planning, and trade assessment in professional baseball.

## 2 Methods

In this study, the following analyses are performed with R (version 3.5.1), a programming language for statistical analysis.

### 2.1 Principal component analysis

Prior to performing principal component analysis, all performance indices were standardized using z-scores, such that each variable had a mean of zero and a standard deviation of one. This standardization was applied to account for differences in scale and units among the 115 performance indices and to ensure that each variable contributed equally to the principal component extraction.

When performing multivariate analysis, the variables may be reduced or selected. In this study, we used PCA. The number of principal components retained in each category was determined based on the cumulative variance explained ratio. Principal components were retained until approximately 70% of the total variance was explained. PCA is a method to synthesize variables known as the principal component score, which best represents the overall variability of a small number of uncorrelated variables from many correlated variables.

Let $X_{n\times p}$ be a dataset comprising *n* individuals and *p* variables. The composite variable is represented by a linear combination of *p* -dimensional data reduced to lower *k* dimensions (k≤p),

$$z_{j}=a_{1,j}x_{1}+a_{2,j}x_{2}+a_{3,j}x_{3}+\cdots +a_{\;p,j}x_{p},\;\;\;(\;j=1,\cdots ,k)$$

For convenience, the coefficient data are denoted by coefficient matrix $A_{\;p\times k}$. The value $Z_{n\times k}=X_{n\times p}A_{\;p\times k}$, which is obtained using the linear combination formula of data $X_{n\times p}$ and $A_{\;p\times k}$ is known as the principal component score.

In PCA, these principal components are obtained under the constraint $\overset{p}{\underset{i=1}{\sum }}a_{i,j}=1$ such that the variance of $z_{j}$ is maximized. Consequently, we arrive at an eigenvalue problem for the variance–covariance matrix of the data $X_{n\times p}$ when focusing on variance and an eigenvalue problem for the correlation coefficient matrix of the data $X_{n\times p}$ when focusing on correlation.

The eigenvectors are the principal components and are equal to the square of the standard deviation of the principal component scores. Principal components with larger values contain more information regarding the original data.

The information of the original variable, which is reduced to principal components, can be confirmed by principal component loadings. The principal component loadings take values from −1 to 1 and represent the correlation with the original variables.

The interpretation and labeling of each principal component were based on the dominant original variables identified through the loading patterns, following standard practices in multivariate sports performance analysis.

### 2.2 Gaussian mixture model

Some data may have a multi-peak distribution with two or more peaks. For such data, a model assuming a unimodal distribution with only one peak, such as a normal distribution, is inappropriate. Instead, a Gaussian mixture model is used, which also assumes a composite of two or more unimodal distributions.

Let $f_{1}(x;\theta_{1}),\ldots ,f_{G}(x;\theta_{G})$ denote the probability density functions of the G normal distributions contained in the arbitrary mixture distribution, and $\pi_{1},\ldots ,\pi_{G}$ denote their mixing ratios.

$\theta_{g}(g=1,\ldots ,G)$ is a vector comprising the parameters contained in $f_{g}(x;\theta_{g})$. In addition, the mixing ratio, $\pi_{1},\ldots ,\pi_{G}$ is assumed to satisfy $0\leq \pi_{g}\leq 1(g=1,\ldots G),\overset{G}{\underset{g=1}{\sum }}\pi_{g}=1$. In this case, the probability (density) function of the mixed normal distribution model is given by:

$$f(x;\theta )=\overset{G}{\underset{g=1}{\sum }}\pi_{g}f_{g}(x;\theta_{g})$$

To estimate the parameters $\theta =(\theta_{1}^{T},\ldots ,\theta_{G}^{T},\pi_{1},\ldots ,\pi_{G-1}{)}^{T}$ in this model, we use the EM algorithm.

The conditional expectation used in Step E of the EM algorithm is given by the following equation:

$$\gamma_{ig}=E(Z_{ig}|x_{i})=Pr(Z_{ig}=1|x_{i})$$

$$Pr(Z_{ig}=1|x_{i})=\frac{\pi_{g}f_{g}(x_{i};\theta_{g})}{\sum_{h=1}^{G}\pi_{h}f_{h}(x_{i};\theta_{h})}$$

Cluster analysis can be performed using a mixture distribution by classifying the i-th observation into the component with the largest estimated value of these equations.

The number of clusters and BIC (7) of the variance–covariance matrix allows the selection of the best model.

Specifically, models with different numbers of clusters were compared using BIC values, and the model with the lowest BIC was selected as the optimal solution, balancing goodness of fitness and model complexity.

### 2.3 Procedure

Using these methods, the following steps are used in the analysis.

① The indices are divided into clusters for each of the eight categories defined by DELTA Inc. In addition, to prevent the cluster from being divided between players who have a good chance of batting and those who do not, we narrow it to 72 players whose wins above replacement (WAR) (11) is 1.0 or higher. The WAR expresses the contribution of a player by comprehensively evaluating hitting, baselining, defense, and pitching based on sabermetrics.
② PCA and cluster analysis were applied to the 72 players narrowed down in ② for each of the eight categories defined by DELTA Corporation.

**Figure 1:**
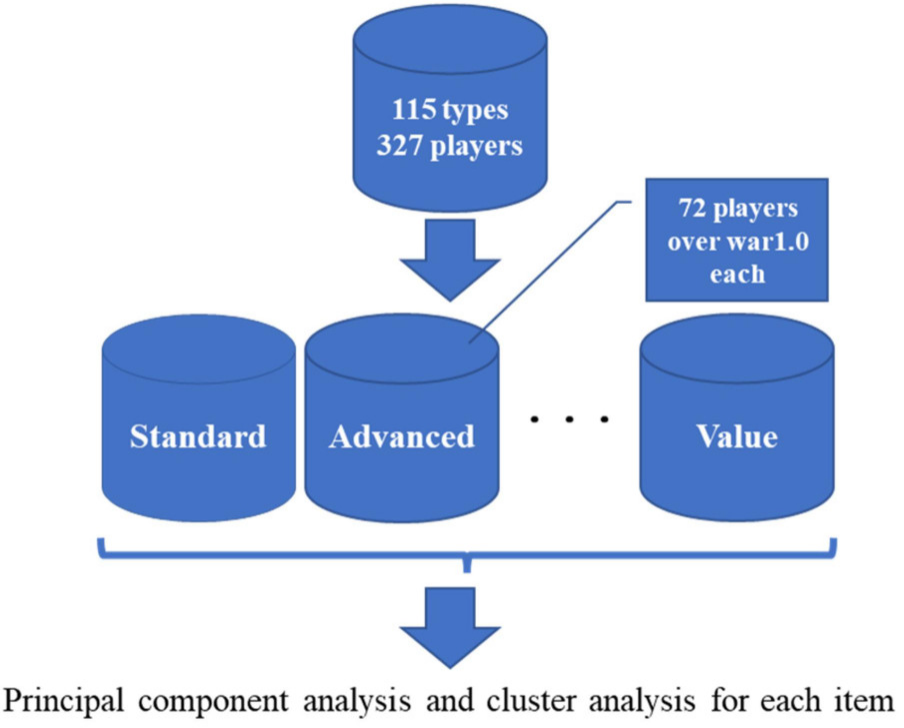
Step ①.

**Figure 2:**
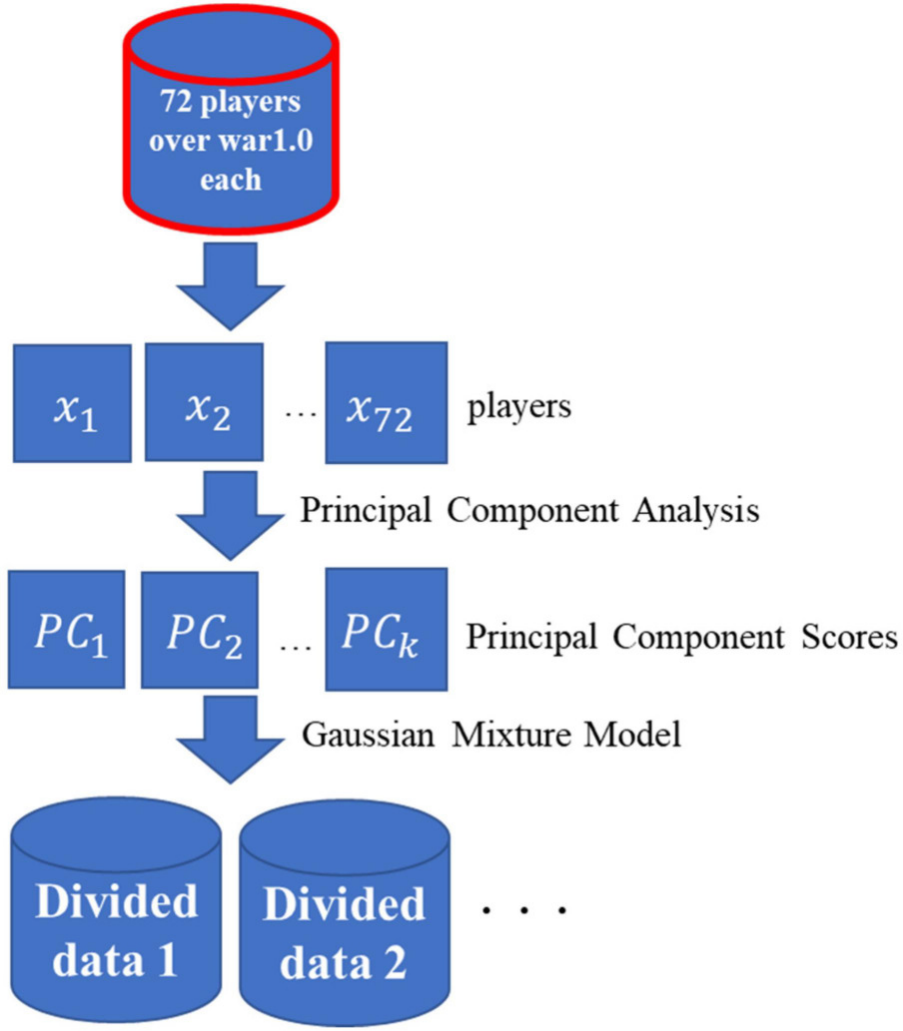
Step ②.

Figures 1, 2 illustrate the flow of the analysis.

## 3 Data

We analyzed 115 different hitting performance indices for 327 fielders who played official games in the 2020 Nippon Professional Baseball (NPB) season. The data were obtained from “1.02 Essence of Baseball,” a professional baseball analytics service provided by DELTA Inc. A detailed description of all indices is provided in the Appendix.

While the official website of the NPB organization provides a limited set of 22 batting indices, the dataset used in this study includes a substantially larger number of performance measures, enabling a more comprehensive multivariate analysis of batting performance.

The batting performance indices were grouped into eight categories based on their conceptual meaning, as summarized in Table 1. These categories include standard statistics, advanced sabermetric indices, batted-ball characteristics, win probability measures, pitch-related data, plate discipline metrics, and value-based indices used in the calculation of wins above replacement (WAR).

**Table 1:** List of DELTA corporation indices.

Category	Summary
Standard	Standard non-sabermetric indices, such as PA (batting average) and AVG (batting average).
Advanced	Common sabermetric indices such as SLG (slugging percentage) and OBP (on-base percentage).
Batted ball	Statistical data on batted balls. Percentage of ground balls and fly balls (GB%, FB%), etc.
Win probability	Indices of winning contribution, such as WPA+, WPA- (the sum of increasing and decreasing win expectancies), etc.
Pitch type	Data on the percentage of pitches thrown and the velocity of pitches, including FAv and CTv (average velocity of straight and cut balls). CB% is the percentage of curveballs thrown.
Pitch value	Data on the percentage of pitches thrown and the velocity of pitches, including FAv and CTv (average velocity of straight and cut balls).
Plate discipline	Data compiled on the batter's pitch selection, such as Contact% (the percentage of batted balls hit by opposing batters as they swing).
Value	A set of indices necessary to calculate WAR.

## 4 Result

The following are the results of the analysis performed on 72 players with a WAR of 1.0 or higher, dividing the index by the eight categories defined by DELTA Inc.

### 4.1 Principal component analysis

**Figure 3:**
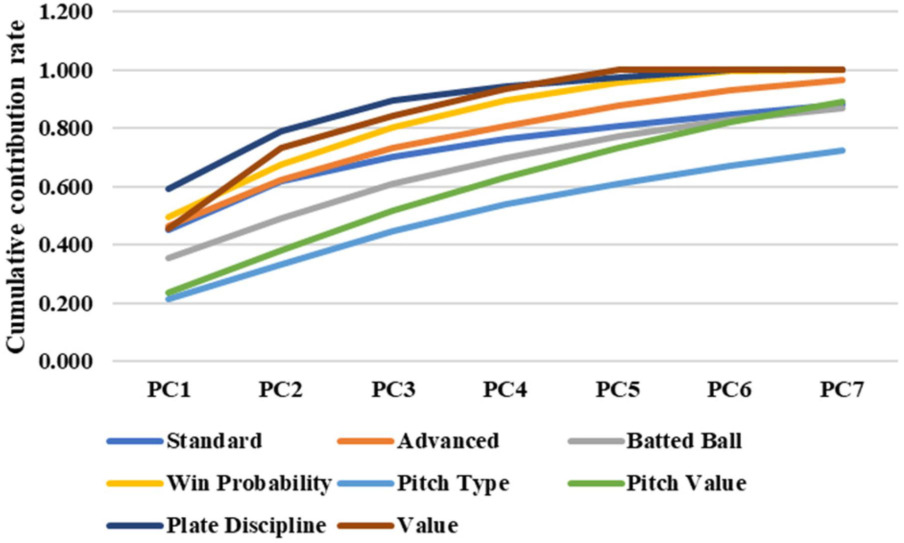
Cumulative contribution rate of each category.

Figure 3 shows the results of the PCA, summarizing the cumulative contribution ratio. The principal components with a cumulative contribution ratio of approximately 70% were selected, as listed in Table 2.

**Table 2:** Number of selected principal components.

Standard	Advanced	Batted ball	Win probability
3	3	4	2
Pitch type	Pitch value	Plate discipline	Value
6	5	2	2

Next, we attempted to interpret the characteristics of the principal components based on their loadings. Tables 3, 4 summarize the top three indices with the highest loadings for the principal components in each category. Only the advanced and win probabilities are shown here as examples.

**Table 3:** Top indices with high loadings (advanced).

Category	PC1	PC2	PC3
Advanced	wOBA	ISO	PA
	wRC+	SLG	BB/K
	OPS	PA	wRC

wOBA, weighted on-base average; ISO, isolated power; PA, plate appearances; wRC+, weighted runs created plus; SLG, slugging percentage; BB/K, walk-to-strikeout ratio; OPS, on base plus slugging; wRC, weighted runs created.

**Table 4:** Top indices with high loadings (Win probability).

Category	PC1	PC2
Win probability	REW	WPA-
	RE24	Clutch
	WPA	PH

REW, run expectancy weighted; WPA−, decrease in win probability added; RE24, run expectancy based on the 24 base–out states; Clutch, clutch performance metric (performance in high-leverage situations); WPA, win probability added.

PH, pinch hitter.

Based on these indices, the characteristics of each principal component can be interpreted based on loading patterns, as summarized in Tables 5, 6.

**Table 5:** Principal component interpretation (advanced).

Category	PC1	PC2	PC3
Advanced	Batting contribution	Slugger	Batting eye

**Table 6:** Principal component interpretation (Win probability).

Category	PC1	PC2
Win probability	Victory contribution	Resilient to chance

For instance, for the first principal component of advanced, the principal component loadings of indices such as wOBA, wRC+, and OPS, which evaluate the contribution of each player to the increase in team runs scored purely by hitting, are high. In other words, the higher the value of the principal component score calculated by the first principal component, the higher is the player's contribution to hitting.

For the second principal component, the principal component loadings of the indices that evaluate long-ball hitting ability, such as ISO and SLG, are high. In other words, the higher the value of the principal component score calculated by the second principal component, the more “hard-hitting” the player is characterized.

For the first principal component of win probability, the principal component loadings of indices such as REW, RE24, and WPA, which evaluate the extent to which each player increases or decreases their expected score in important situations that lead to victory, are high. In other words, the higher the value of the principal component score calculated by the first principal component, the higher is the player's contribution to winning.

For the second principal component, the principal component loadings for WPA, which represents the extent to which a player has reduced his expected score compared to the average hitter in the league, are high. In contrast, WPA- has a higher clutch, which is different in that it is a comparison to the average hitter in the league, but to the player himself; thus, the second principal component is characterized as “good at chances.”

### 4.2 Gaussian mixture model

A Gaussian mixture model was constructed using principal component scores. Table 7 lists the number of clusters with the highest BIC values for each category.

**Table 7:** Number of clusters with high BIC.

Standard	Advanced	Batted ball	Win probability
3	2	2	2
Pitch type	Pitch value	Plate discipline	Value
2	2	1	2

Table 8 summarizes the results of classifying players according to the number of clusters in Table 7. Three representative players were selected, based on the characteristics of each cluster. The “number” indicates the cluster type, and the parentheses after the surname indicate the team's name (abbreviation) listed below.

**Table 8:** Players by cluster.

Category	No	Players		
Standard	1	Yanagita(H)	Murakami(S)	Suzuki(C)
	2	Shuto(H)	Genda(L)	Chikamoto(T)
	3	Masuda(G)	Ogo(E)	Kawashima(H)
Advanced	1	Nakashima(F)	Kyoda(D)	Tatsumi(E)
	2	Yanagita(H)	Murakami(S)	Asamura(E)
Batted ball	1	Kawashima(H)	Yoshida(Bs)	Tatsumi(E)
	2	Masuda(G)	Kinoshita(D)	Wada(M)
Win probability	1	Yanagita(H)	Asamura(L)	Yoshida(Bs)
	2	Nakashima(F)	Wada(M)	Kai(H)
Pitch type	1	Wada(M)	Nakashima(F)	Moya(Bs)
	2	Shiomi(S)	Maru(G)	Chono(C)
Pitch value	1	Yanagita(H)	Murakami(S)	Kondo(F)
	2	Kai(H)	Genda(L)	Tatsumi(E)
Value	1	Yanagita(H)	Murakami(S)	Suzuki(C)
	2	Nakashima(F)	Wada(M)	Fushimi(Bs)

C: Hiroshima Toyo Carp

D: Chunichi Dragons

DB: Yokohama DeNA BayStars

G: Yomiuri Giants

S: Tokyo Yakult Swallows

T: Hanshin Tigers

Bs: Orix Buffaloes

E: Tohoku Rakuten Golden Eagles

F: Hokkaido Nippon-Ham Fighters

H: Fukuoka Softbank Hawks

L: Saitama Seibu Lions

M: Chiba Lotte Marines

If the player is advanced, they belong to Cluster 2, as shown in Figure 10 in the Appendix, with the highest first principal component score. Yanagita, Murakami, and Asamura achieved the highest scores for the first principal component in Cluster 2.

Conversely, players with low principal component scores belonged to Cluster 1. Nakashima, Kyoda, and Tatsumi had the lowest principal component scores in this cluster.

In advanced, the scatter plots are shown in Figures 10–12 in the Appendix, which show the cluster divisions by color.

As advanced software was used up to the third principal component in its analysis, three scatter plots were created. Figure 10 shows the principal component scores calculated using the first principal component on the horizontal axis, and those calculated using the second principal component on the vertical axis. Similarly, Figure 11 shows the second principal component scores on the horizontal axis and the third principal component scores on the vertical axis. Figure 12 shows the first and third principal component scores on the horizontal and vertical axes, respectively.

In Figures 11, 12, the players with high and low scores on the third principal component are divided into clusters. Because the third principal component was “good pitch selection,” it can be interpreted that the clusters were divided between players with good pitch selection and those with poor pitch selection.

Figure 13 shows a scatterplot of the win probability clusters, indicating the cluster division by color.

The horizontal axis is the first principal component score, and the vertical axis is the second principal component score. The players were divided into clusters with high and low scores on the first principal component. Because the first principal component was “contribution to victory,” it can be interpreted that the clusters are divided into squads of players with high contribution to victory and those with low contribution to victory. For instance, Yanagita from SoftBank and Nakashima from Nippon-Ham are placed in Clusters 1 and 2, respectively.

Tables 9–15 summarize the results of calculating the mean principal component scores for each category cluster.

**Table 9:** Principal component score average (standard).

Cluster number	PC1	PC2	PC3
1	2.905	−1.297	−0.262
2	−0.002	1.665	0.757
3	−3.034	−0.687	−0.655

**Table 10:** Principal component score average (advanced).

Cluster number	PC1	PC2	PC3
1	−1.054	−0.143	1.010
2	0.997	0.135	−0.956

**Table 11:** Principal component score average (batted ball).

Cluster number	PC1	PC2
1	0.640	0.730
2	−0.640	−0.730
Cluster Number	PC3	PC4
1	0.186	−0.208
2	−0.186	0.208

**Table 12:** Principal component score average (Win probability).

Cluster number	PC1	PC2
1	1.328	−0.462
2	−1.859	0.647

**Table 13:** Principal component score average (pitch type).

Cluster number	PC1	PC2	PC3
1	−0.199	−0.175	0.231
	PC4	PC5	PC6
	0.332	0.292	−0.385
2	PC1	PC2	PC3
	0.273	−0.273	−0.005
	PC4	PC5	PC6
	−0.455	0.456	0.008

**Table 14:** Principal component score average (pitch value).

Cluster number	PC1	PC2	PC3	PC4	PC5
1	0.411	−0.522	0.122	0.072	−0.292
2	−0.998	1.269	−0.297	−0.175	0.709

**Table 15:** Principal component score average (value).

Cluster number	PC1	PC2
1	0.844	0.137
2	−1.687	−0.274

The cluster means for each category corresponds to the scatterplot. For instance, in advanced category, Cluster 2 has a higher average first principal component score than Cluster 1. Figure 10 shows that the players belonging to Cluster 2 are closer to the first quadrant. For win probability, Cluster 1 has a higher average first principal component score than Cluster 2. Figure 13 shows that the players belonging to Cluster 1 are closer to Quadrants 1 and 4.

## 5 Discussion

### 5.1 Possibility to be an alternative player

Players classified into the same cluster share similar latent performance characteristics as summarized by the principal components derived from multiple hitting indices. Such similarity suggests that these players may fulfill comparable offensive roles within a team, making them potential substitutes for one another. Unlike simple comparisons based on individual statistics, this framework captures multidimensional performance profiles, allowing for a more structured interpretation of player similarity.

**Figure 4:**
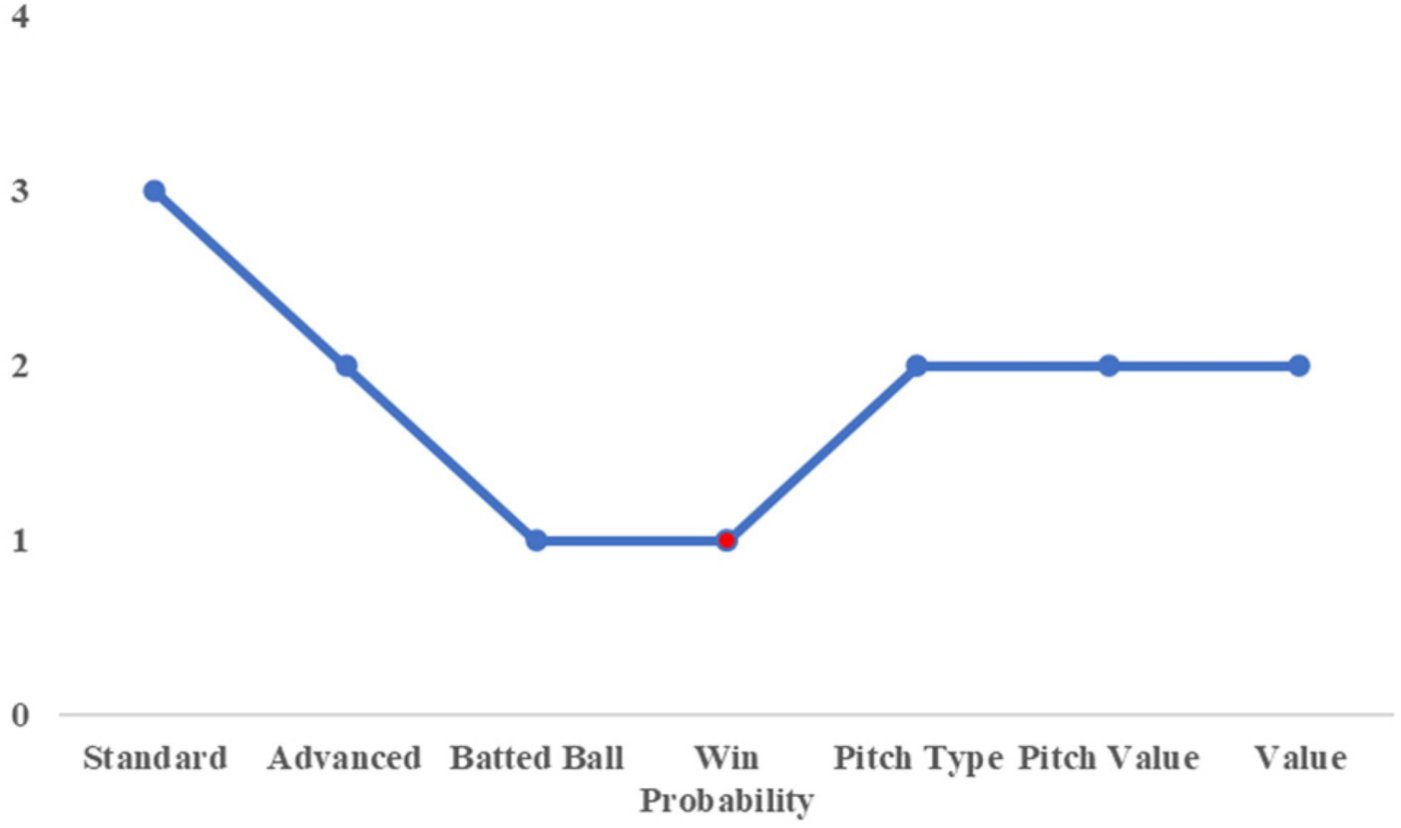
Cluster number by category (for yamada).

**Figure 5:**
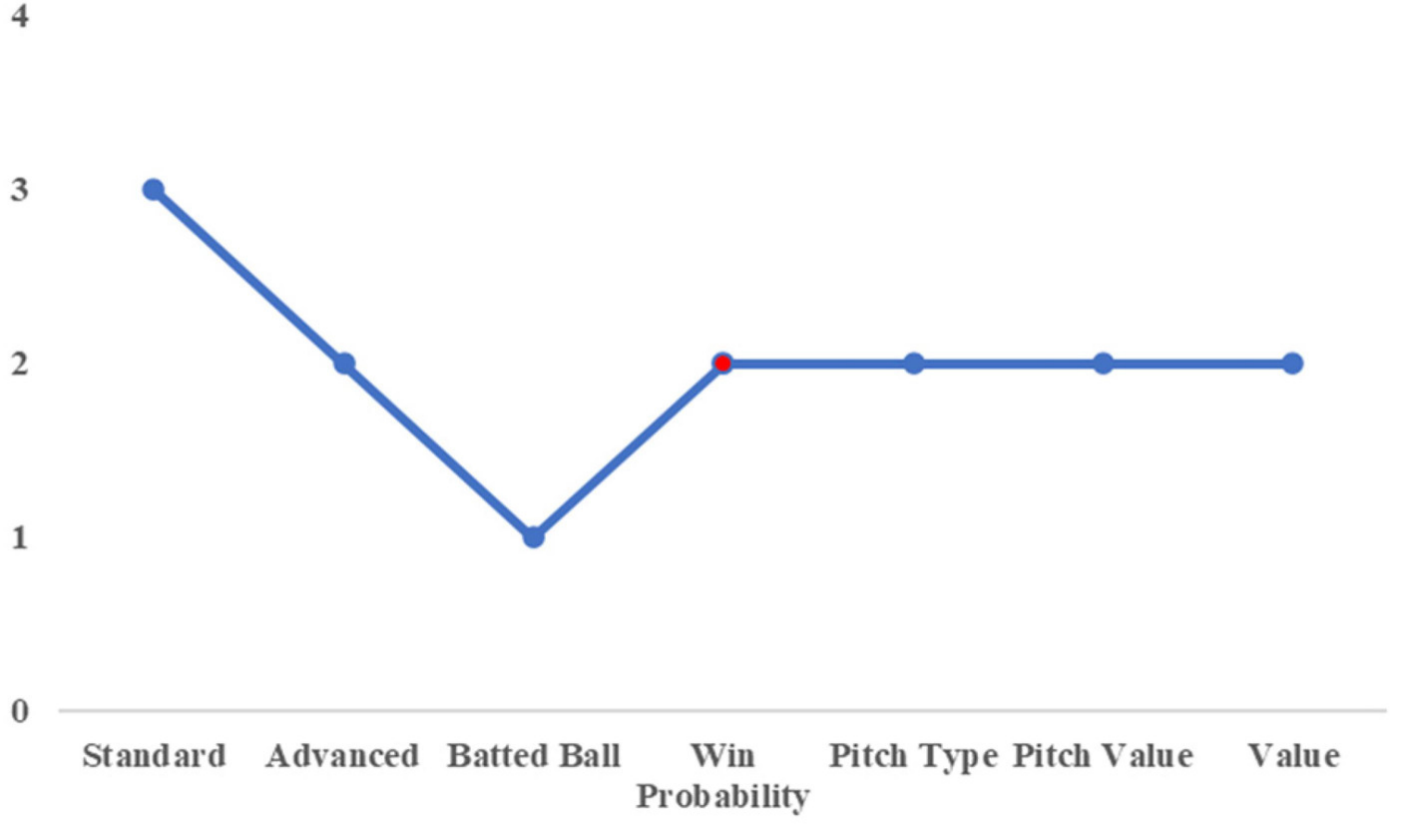
Cluster number by category (for shiomi).

To illustrate this point, we first consider the case of Yamada and Shiomi of the Tokyo Yakult Swallows. Figures 4, 5 show the cluster assignments by category for both players. Except for the win probability category, they were classified into the same clusters across most performance dimensions. In the advanced statistics category, both players belonged to Cluster 2, characterized by higher scores on the first and second principal components and lower scores on the third component compared with Cluster 1.

The first principal component in this category represents overall batting contribution to team run production, while the second component reflects power-related attributes, such as batted-ball distance. The third component is associated with pitch selection quality, with lower scores indicating less selective plate discipline. From this perspective, Yamada and Shiomi can be interpreted as power-oriented hitters with high run contributions, albeit with relatively weaker pitch selection. This shared latent profile suggests functional similarity in their offensive roles.

This interpretation is consistent with actual team decisions. In the 2021 season, when Yamada—who had been batting in the cleanup position—was unavailable due to injury, Shiomi was promoted from the sixth spot to the cleanup role. Their subsequent performance further supports this substitution. Yamada recorded a WAR of 5.9 in 2021, while Shiomi recorded a WAR of 3.7, the second highest on the team. Although their absolute contributions differed, Shiomi emerged as the most suitable replacement among available players. This example demonstrates that the proposed clustering framework can identify plausible substitutes based on shared performance characteristics rather than surface-level statistics alone.

**Figure 6:**
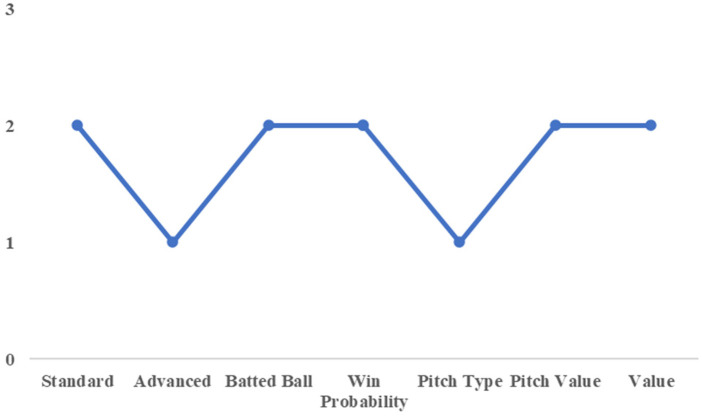
Cluster number by category (for adachi).

**Figure 7:**
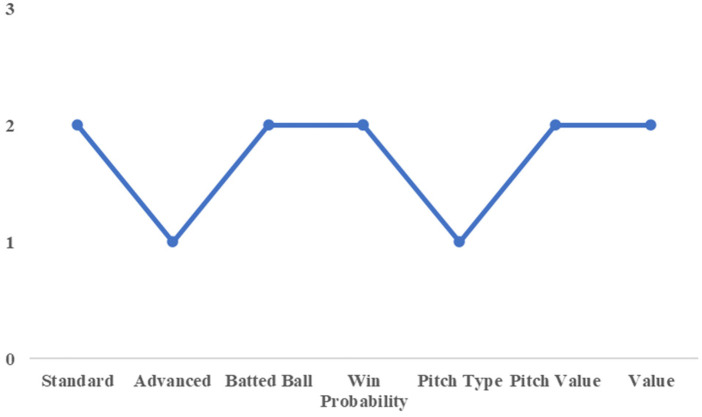
Cluster number by category (for fukuda).

A similar pattern is observed for Adachi and Fukuda of the Orix Buffaloes. As shown in Figures 6, 7, these players were classified into the same clusters across all performance categories. In the win probability category, both belonged to Cluster 2, characterized by lower scores on the first principal component and higher scores on the second component. The first component reflects overall impact on win expectancy, while the second captures performance in high-leverage situations relative to a player's baseline performance.

These results suggest that Adachi and Fukuda share a similar profile as hitters who tend to perform relatively well in critical situations, despite having below-average overall win contributions. In the 2021 season, the team employed both players concurrently, with Adachi reassigned to second base according to team policy. Their WAR values were identical at 1.9, indicating nearly equivalent contributions to team success. Although they were not strictly interchangeable in terms of defensive position, the simultaneous use of two players with comparable cluster characteristics reflects a reasonable personnel strategy aligned with the implications of this study.

Taken together, these cases illustrate that players within the same cluster tend to exhibit similar offensive roles and contribution patterns, supporting the interpretation that such players can serve as substitutes in actual team management contexts.

### 5.2 Possibility of trade

In contrast to substitution scenarios, trades often involve the exchange of players with distinct performance profiles to rebalance team composition or address specific strategic needs. Within the proposed framework, players belonging to different clusters can therefore be interpreted as representing different offensive roles or player types, making them plausible candidates for trade.

**Figure 8:**
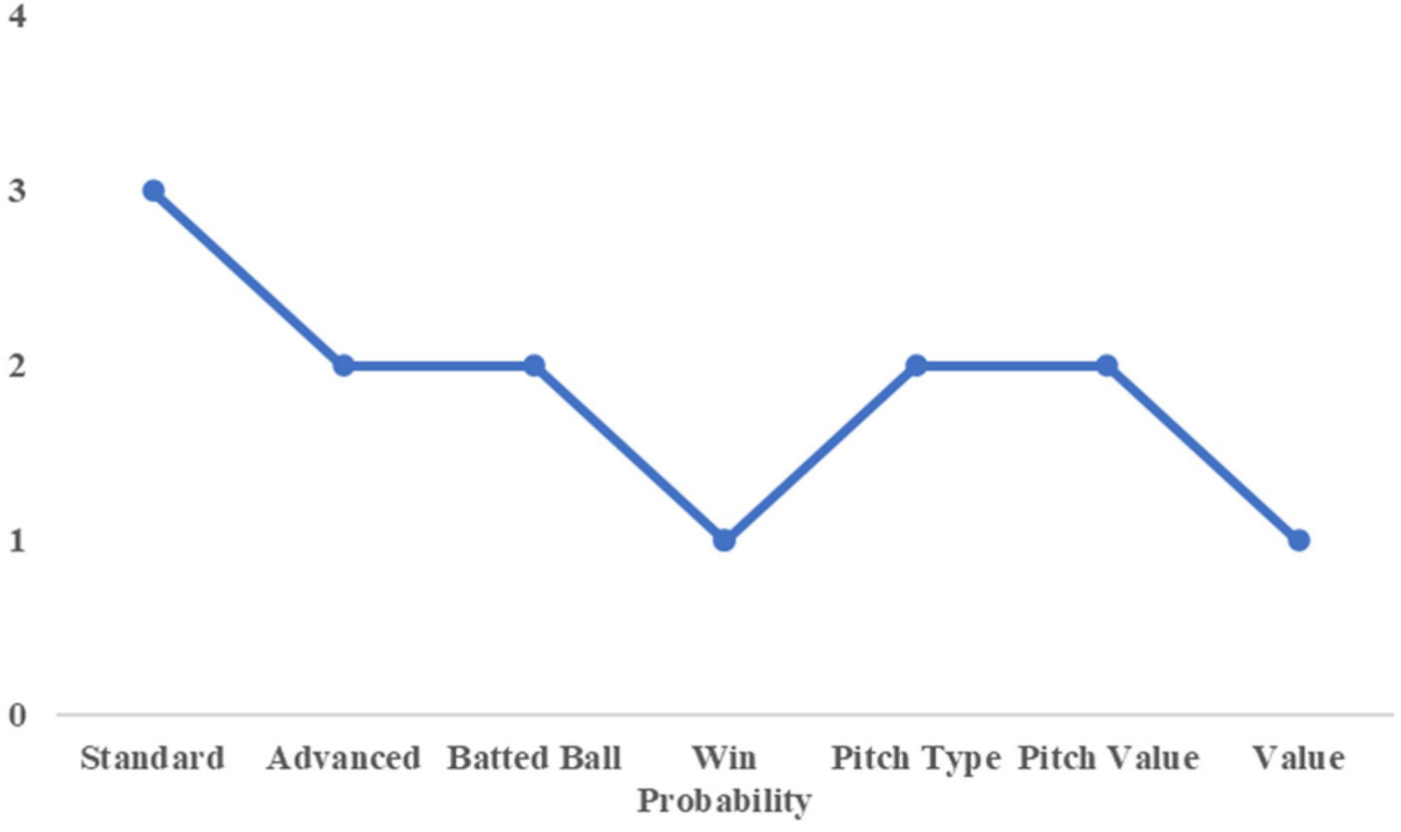
Cluster number by category (for chono).

**Figure 9:**
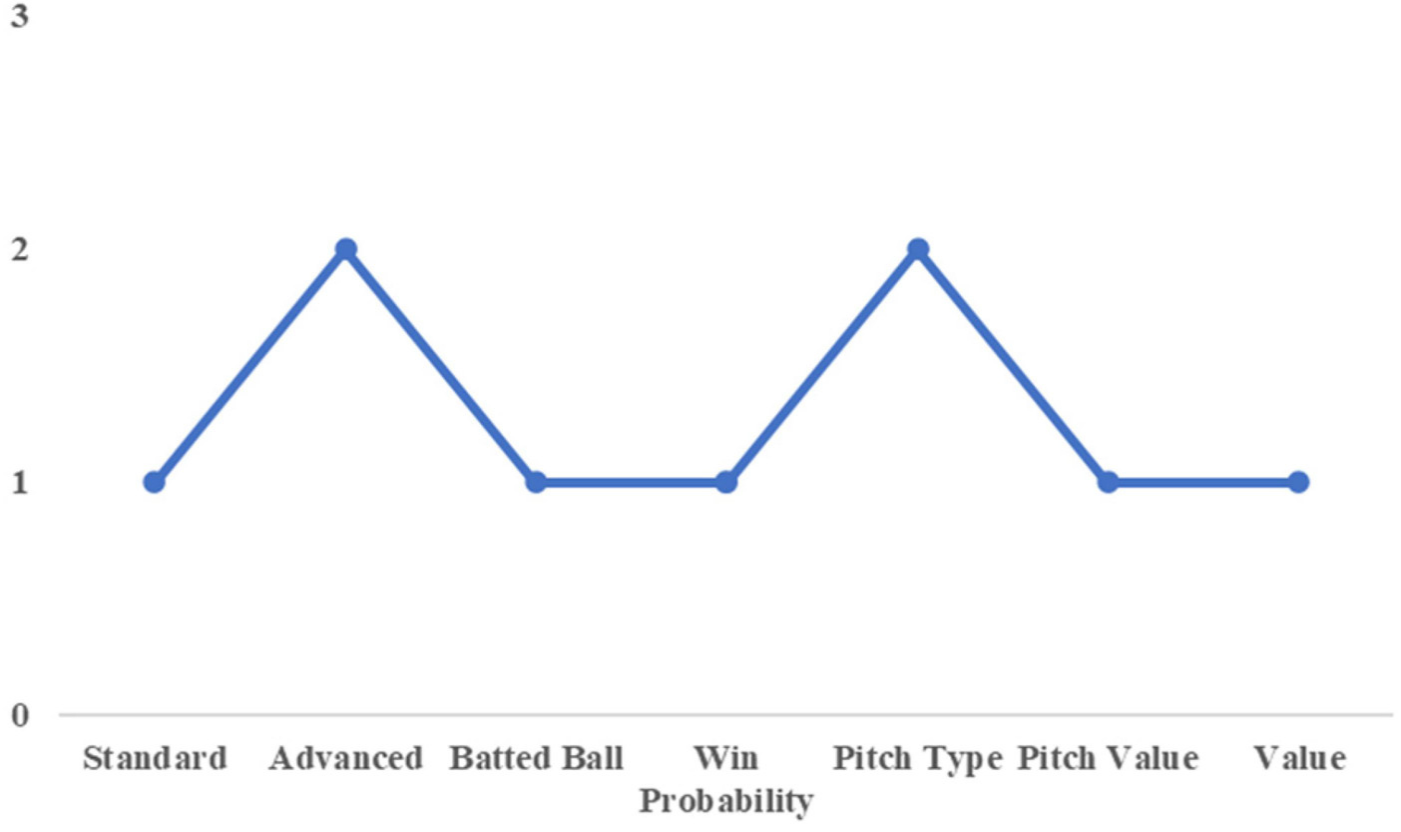
Cluster number by category (for Maru).

As an illustrative example, we examine the trade involving Chono and Maru prior to the 2019 season, when Chono moved from the Yomiuri Giants and Maru joined the Giants from the Hiroshima Toyo Carp. Figures 8, 9 show that these players were classified into different clusters in several categories, including Standard, Batted Ball, and Pitch Value. This indicates clear differences in their underlying offensive characteristics as captured by the principal components.

**Figure 10:**
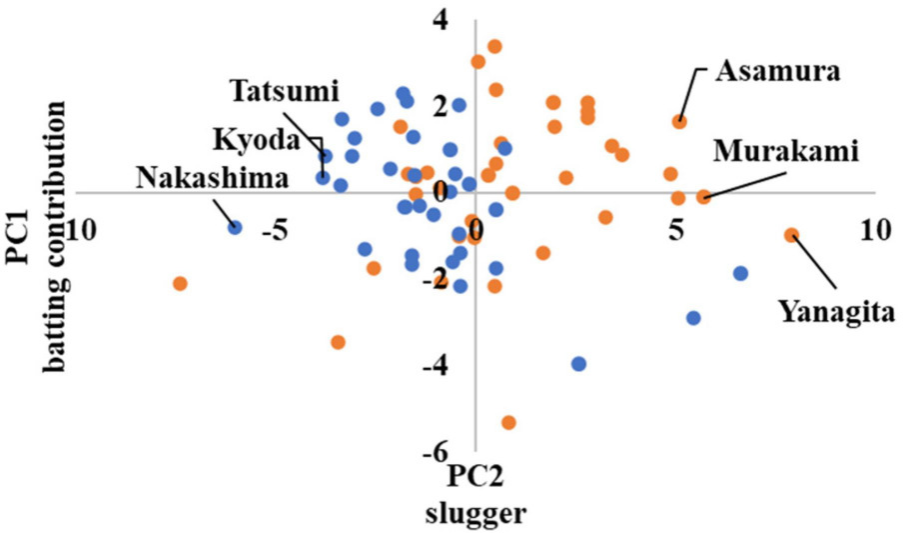
Scatter plots by advanced cluster ①.

**Figure 11:**
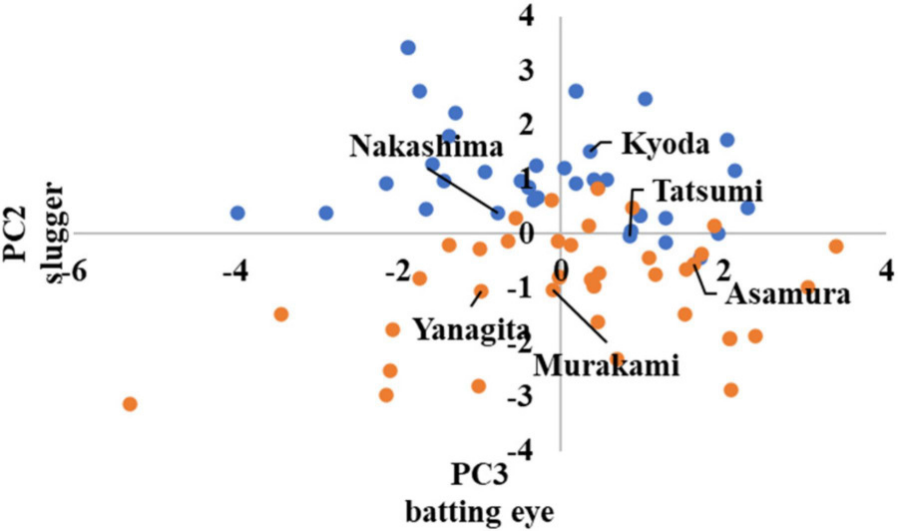
Scatter plots by advanced cluster ②.

**Figure 12:**
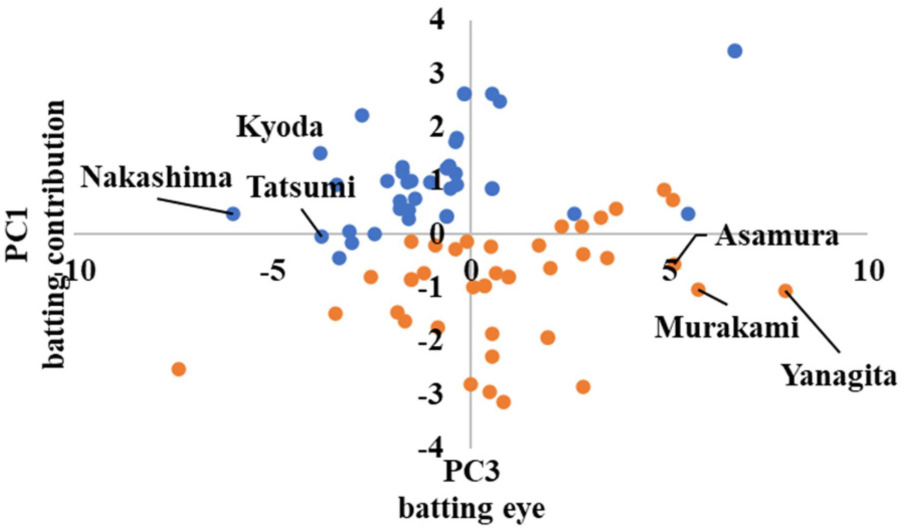
Scatter plots by advanced cluster ③.

**Figure 13:**
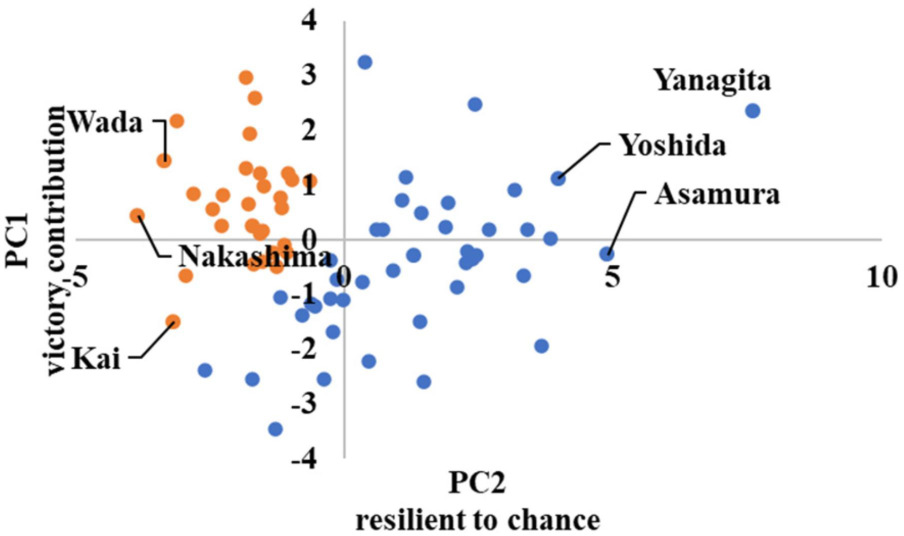
Scatter plots by win probability cluster.

Their subsequent performances further support this interpretation. In the 2021 season, Maru recorded a WAR of 3.9, while Chono recorded a WAR of −0.2. From the perspective of on-field value, this outcome suggests that the Giants benefited more from acquiring Maru. Unlike the substitution examples discussed earlier, this case illustrates a trade involving players with clearly distinct performance profiles, consistent with the notion that trades often aim to exchange different types of players rather than similar ones.

This example highlights that cluster dissimilarity may serve as a useful indicator for identifying players who can provide contrasting contributions when exchanged between teams, complementing outcome-based evaluations such as WAR.

### 5.3 Implications and limitations

Overall, the results suggest that clustering based on latent performance characteristics can provide a structured perspective on player similarity and dissimilarity, with potential applications to substitution planning and trade assessment. By reducing high-dimensional performance data into interpretable components, the proposed framework enables comparisons that extend beyond individual statistics.

The cluster-specific performance profiles may also inform player development strategies. For example, training programs could be tailored to cluster-defined strengths and weaknesses, enabling more targeted skill development and role optimization within teams.

Several limitations should be acknowledged. First, the analysis is based on a single season of data, which may not fully capture variability in player performance across seasons. Second, the present study focuses exclusively on hitting-related indices and does not account for defensive performance or positional constraints. Future research could extend this framework by incorporating multi-season data, defensive metrics, and additional contextual factors to further enhance its applicability to team decision-making.

## 6 Conclusion

This study proposed a framework for classifying Japanese professional baseball fielders based on latent hitting performance characteristics derived from a comprehensive set of indices. By applying principal component analysis and Gaussian mixture model–based clustering to 115 hitting indices across eight performance categories, we demonstrated that high-dimensional batting data can be condensed into interpretable structures that reveal meaningful player similarities and differences.

The results suggest that players classified within the same cluster tend to share comparable offensive roles and may serve as potential substitutes, while players belonging to different clusters represent distinct performance profiles relevant to trade decisions. In this sense, the proposed framework provides a structured, data-driven perspective on player similarity that complements outcome-based evaluations such as WAR, and offers practical insights for roster construction, substitution planning, and trade assessment in professional baseball.

Despite the limitations of using single-season data and focusing exclusively on hitting-related indices, the present study demonstrates that integrating principal component analysis with Gaussian mixture model–based clustering provides a meaningful and interpretable framework for classifying professional baseball players.

Future research will extend this framework to multi-season datasets, incorporate defensive performance and positional constraints, and apply the approach to pitchers to further enhance their robustness and practical applicability.

## Data Availability

The datasets presented in this study can be found in online repositories. The names of the repository/repositories and accession number(s) can be found in the article/Supplementary Material.

## Appendix 1

About sabermetrics terminology.

The indices and scatter plots related to the sabermetrics that appear in the text are as follows:.

Batting.

Represents the offensive evaluation component of WAR.

BB/K.

Walks per strikeout. Indicates the ratio of walks to strikeouts.

CB%.

Percentage of curveballs faced.

Clutch.

Performance in crucial situations compared to overall performance.

Hard%.

Percentage of batted balls recorded at 95 miles per hour or higher.

IFH%.

Infield hits per ground ball. Proportion of infield hits to ground balls.

ISO.

Isolated power (Slugging percentage minus batting average). Indicator of a hitter's power.

K%.

Strikeout per plate appearance. Indicates the strikeout rate.

OPS.

On base plus slugging. A metric of overall batting effectiveness. Higher values suggest a greater contribution to scoring per plate appearance.

PA.

Plate appearances.

PH.

Pinch hit appearances.

RBI.

Runs batted In.

RE24.

Measures a player's contribution based on changes in run expectancy.

Replacement.

Represents the replacement level comparative value in WAR.

REW.

Wins derived from the total change in run expectancy calculated by RE24.

SLG.

Slugging percentage (Total bases per at-bat). Measures average bases earned per at-bat.

SLv.

Average velocity of sinkers faced.

WAR.

Wins above replacement. Comprehensive measures of a player's contribution through batting, fielding, and baserunning.

wCB.

Total change in runs due to curveballs.

wCB/C.

Change in runs per 100 curveballs faced.

wOBA.

Weighted on-base average. Measures how much the contribution of a player contributes to team scoring per plate appearance.

WPA.

Win probability added. Measures a player's contribution to changing the team's win expectancy.

WPA-.

Total decrease in win expectancy.

WPA+.

Total increase in win expectancy.

wRC.

Weighted runs created. Total offensive runs contributed, adjusted for league and park factors.

wRC+.

Adjusted wRC comparing a player's wRC to league average, takconsidering park factors into account. wRC.

## Appendix 2

Scatter plots of principal component scores

Below are scatterplots with principal component scores that are reduced from “advanced” and “win probability” indices.
