# Correction: Classification of fielders in nippon professional baseball using a Gaussian mixture clustering model

**DOI:** 10.3389/fspor.2026.1823801

**Published:** 2026-05-07

**Authors:** Taishi Oda, Nobuyoshi Hirotsu

**Affiliations:** Graduate School of Health and Sports Science, Juntendo University, Bunkyō, Japan

**Keywords:** baseball, cluster analysis, Gaussian mixture model, principal components analysis, sabermetrics

**A Correction on**
Classification of fielders in nippon professional baseball using a Gaussian mixture clustering model By Oda T and Hirotsu N (2026). Front. Sports Act. Living 8:1612463. doi: 10.3389/fspor.2026.1612463

Reference 3 was erroneously written as:

Miyamoto S. *Introduction to Cluster Analysis: Theory and Applications of Fuzzy Clustering*. Boca Raton: Morikita Publishing Co., Ltd. (1999). p. 13–105.

It should be:

Miyamoto S. *Introduction to Cluster Analysis: Theory and Applications of Fuzzy Clustering*. Tokyo: Morikita Publishing Co., Ltd. (1999). p. 13–105.

The original version of this article has been updated.

